# Severe and disseminated atypical mycobacteriosis of the skin under immunosuppression

**DOI:** 10.1111/ddg.70016x

**Published:** 2026-01-19

**Authors:** Veronika Zenderowski, Laura Schreieder, Mark Berneburg, Dennis Niebel, Sebastian Haferkamp, Sigrid Karrer, Konstantin Drexler

**Affiliations:** ^1^ Klinik und Poliklinik für Dedrmatologie Universitätsklinikum Regensburg, Deutschland

**Keywords:** Atypical mycobacteriosis, Crohn's disease, immunosuppression, TNF‐alpha

Dear Editors,

Atypical mycobacterioses of the skin are rare infections that occur predominantly in immunocompromised patients. ^1^ Because of their variable clinical presentation, they are difficult to diagnose. We report on a patient with Crohn's disease who was treated with adalimumab and developed a disseminated *Mycobacterium marinum* infection. The skin lesions were initially interpreted as a possible drug reaction or cutaneous manifestation of Crohn's disease. This illustrates the diagnostic challenge.

Another patient was presented by the rheumatology department for dermatological co‐evaluation due to newly occurring, painful skin changes. The patient had been suffering from Crohn's disease for 13 years, for which he was originally treated with methotrexate (MTX). Due to the insufficient efficacy of this therapy, treatment was switched to adalimumab around a year ago. Shortly afterwards, the patient developed painful skin changes on his hands for the first time. Due to inadequate symptom control of the bowel disease, treatment was switched to infliximab, whereupon the skin condition deteriorated further. Treatment with prednisolone also led to an increasing spread of the lesions, which eventually affected the entire integument. The patient worked in green space maintenance and privately maintained an aquarium, stocked with cold‐water fish.

Clinical inspection revealed disseminated, sharply defined, livid‐erythematous nodules up to several centimeters in size over the entire integument. There were also pinhead‐sized erythematous papules and plaques several centimeters in diameter, some of which were clearly overheated (Figure 1a‐c). The arms and legs also showed a sporotrichoid pattern of spread with the efflorescences arranged along the lymphatic channels.

**FIGURE 1:**
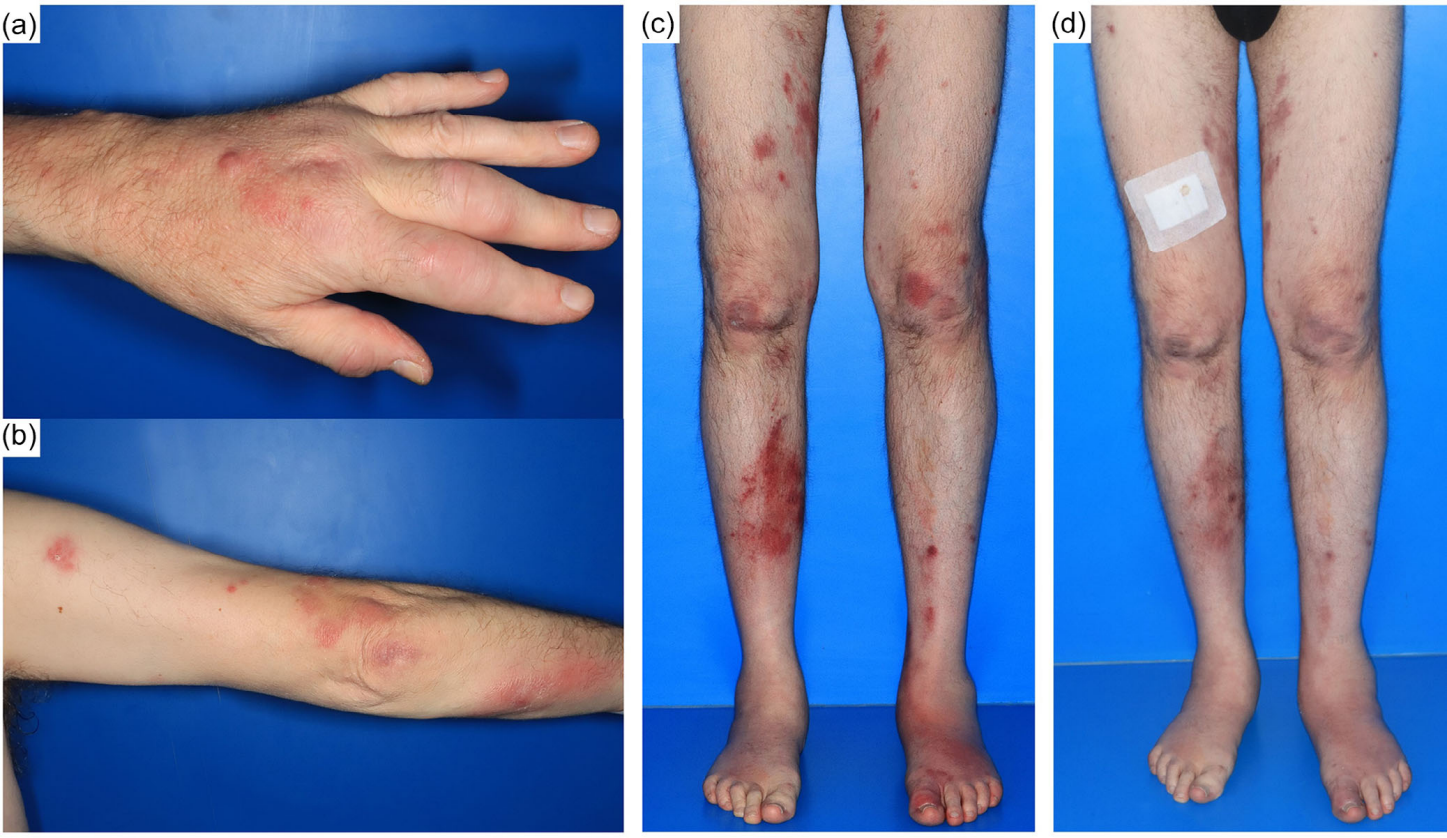
(a) Onset of nodule spread on the hand used for cleaning the aquarium (May 2023). (b,c) Initial sporotrichoid spread on the arm, followed by involvement of the entire integument (May 2023). (d) Marked improvement of the skin lesions approximately four weeks after initiation of therapy (June 2023).

The differential diagnosis included cutaneous manifestations of Crohn's disease, adverse drug reactions or infections. A spindle biopsy from the thigh showed a pronounced lymphohistiocytic infiltrate in the H&E staining (Figure 2a,b). Immunohistochemical examination detected numerous CD68‐positive macrophages in the adipose tissue (Figure 2c). Ziehl‐Neelsen staining remained negative (Figure 2d).

**FIGURE 2:**
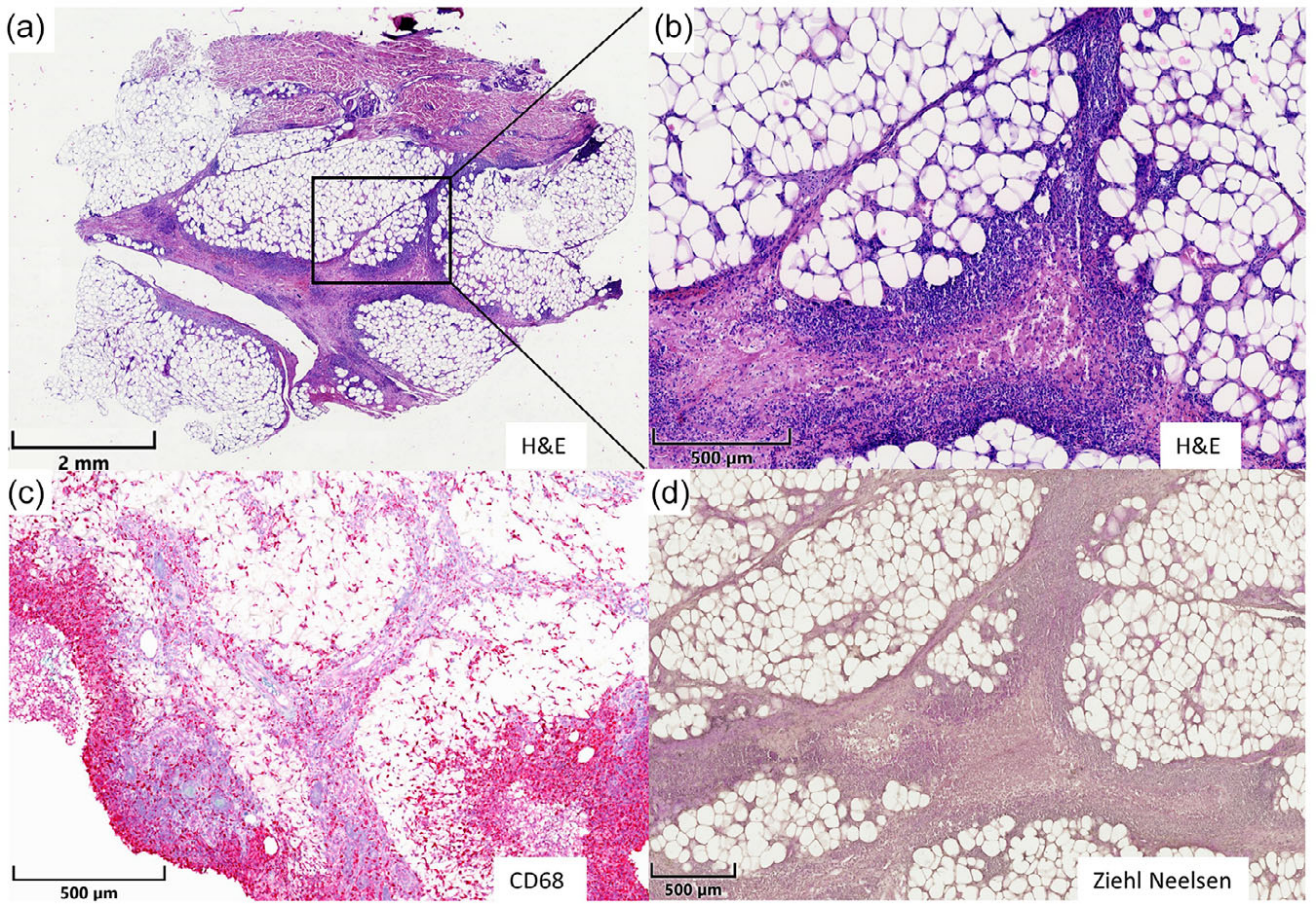
(a) Overview of a thigh biopsy showing a lymphocytic infiltrate within the interlobular septa of subcutaneous fat (H&E, overview). (b) Higher magnification demonstrates a mixed, predominantly lymphocytic infiltrate between the fat lobules (H&E). (c) Numerous CD68‐positive macrophages (CD68). (d) No acid‐fast bacilli detected (Ziehl‐Neelsen).

Microbiological detection of *M. marinum* was successful both by PCR and culture. In the liquid nutrient medium, the culture remained negative (detection limit 10‐100 CFU/ml). *M. marinum* could be cultured on a solid nutrient medium. Resistance testing by microdilution according to the CLSI revealed an MIC of 1.0 mg/L for rifampicin (sensitive) and 2.0 mg/L for ethambutol (no interpretation according to EUCAST 11.0).

After an interdisciplinary consultation, triple therapy with azithromycin, ethambutol, and rifabutin was carried out over a period of eleven months. This treatment required close monitoring: Azithromycin carries the risk of gastrointestinal complaints, hearing impairment and QT time prolongation; ethambutol can cause optic neuritis; rifabutin is associated with hepatotoxicity, uveitis and myelosuppression.^2^ After improvement of the clinical condition, ethambutol was discontinued and dual therapy with azithromycin and rifabutin was continued. Under this treatment, both the skin lesions and the associated pain improved (Figure 1d). Temporary hearing loss and hematuria occurred during the course of treatment, presumably due to the interaction of azithromycin with a DOAC, but both conditions were reversible.

Non‐tuberculous mycobacteria (NTM) include all Mycobacterium species except *M. tuberculosis* complex and *M. leprae*. Although NTM generally have a lower pathogenicity, they can cause a variety of clinical pictures. The skin is the second most common site of manifestation after the lungs.^3^ This is a very heterogeneous group of pathogens, in some cases with substantial differences in diagnostic clarification, resistance profiles and treatment options. *M. marinum* is a special representative in this context, known primarily as a typical pathogen of cutaneous infections, while other NTM, such as *M. fortuitum*, *M. kansasii*, *or M. ulcerans*, have different clinical patterns. *Mycobacterium fortuitum* mainly causes abscesses and chronic wounds, *M. kansasii* is predominantly associated with pulmonary infections, while *M. ulcerans* causes extensive ulcerative lesions (Buruli ulcer) due to the toxin mycolactone.^1^

Infections with *M. marinum* are typically caused by contact of pre‐existing skin lesions with contaminated water. Clinically, purple papules, plaques or nodules occur preferentially on the extremities, often in a sporotrichoid arrangement.^4^ In our case, the immunosuppression led to an unusually pronounced dissemination, so a hematogenous spread must also be assumed.

Diagnosis is based on history and inspection as well as histopathological examination of a lesion and cultivation or detection of the bacterium by PCR. Histologically, a characteristic granulomatous and suppurative inflammatory process can be seen in the dermis. In the present case, Ziehl‐Neelsen staining failed to detect acid‐fast rods. This often occurs in *M. marinum* infections due to the low sensitivity of the method. Cultivation in culture with subsequent resistance determination is still the diagnostic gold standard. Specifying the suspected diagnosis in the request is crucial in order to ensure the special culture conditions required for mycobacteria and to avoid false negative results.^5^ In addition, specific PCR tests can be used to identify and differentiate non‐tuberculous mycobacteria (NTM), providing significantly faster results compared to culture.^6^ The differential diagnosis includes sporotrichosis, leishmaniasis, fungal infections and granulomatous diseases such as sarcoidosis, foreign body reactions or tuberculosis.^1^, ^6^

The choice of therapy is based on various factors, including the NTM subtype, the extent of the lesions and the patient's immune status. In mild cases, monotherapy with clarithromycin, minocycline, doxycycline, or trimethoprim/sulfamethoxazole may be effective. Severe cases such as this often require combination therapies including rifampicin, ethambutol, macrolides, and trimethoprim/sulfamethoxazole.^2^, ^4^ Despite the persistence of individual lesions, significant clinical improvement was achieved in the present case, demonstrating the efficacy of the therapy.

Cutaneous side effects including infectious complications have been described with adalimumab. In addition to maculopapular rashes or psoriasiform lesions, the immunosuppressive effect can increase susceptibility to infections with atypical mycobacteria.^7^ Although it remains unclear whether the use of adalimumab promoted the infection in the case presented here, it seems likely. Therapeutically, alternative immunosuppressants such as IL‐23 blockers (e.g. guselkumab) or JAK inhibitors (e.g. upadacitinib) could be considered,^8^, ^9^ as they are less frequently associated with NTM infections.^10^

This case illustrates the importance of early differential diagnosis and targeted microbiological diagnostics for the management of atypical mycobacteriosis, especially in immunocompromised patients.

## CONFLICT OF INTEREST STATEMENT

None.
